# Medical financial hardship between young adult cancer survivors and matched individuals without cancer in the United States

**DOI:** 10.1093/jncics/pkae007

**Published:** 2024-02-14

**Authors:** Lihua Li, Donglan Zhang, Yan Li, Mayuri Jain, Xingyu Lin, Rebecca Hu, Junxiu Liu, Janani Thapa, Lan Mu, Zhuo Chen, Bian Liu, José A Pagán

**Affiliations:** Department of Population Health Science and Policy, Icahn School of Medicine at Mount Sinai, New York, NY, USA; Institute for Health Care Delivery Science, Icahn School of Medicine at Mount Sinai, New York, NY, USA; The Tisch Cancer Institute, Icahn School of Medicine at Mount Sinai, New York, NY, USA; Department of Geriatrics and Palliative Medicine, Icahn School of Medicine at Mount Sinai, New York, NY, USA; Division of Health Services Research, Department of Foundations of Medicine, NYU Long Island School of Medicine, Mineola, NY, USA; Department of Population Health Science and Policy, Icahn School of Medicine at Mount Sinai, New York, NY, USA; School of Public Health, Shanghai Jiao Tong University School of Medicine, Shanghai, China; Department of Population Health Science and Policy, Icahn School of Medicine at Mount Sinai, New York, NY, USA; Institute for Health Care Delivery Science, Icahn School of Medicine at Mount Sinai, New York, NY, USA; The Tisch Cancer Institute, Icahn School of Medicine at Mount Sinai, New York, NY, USA; Department of Statistical and Actuarial Sciences, University of Western Ontario, London, ON, Canada; Department of Molecular and Cellular Biology, University of California, Berkeley, Berkeley, CA, USA; Department of Population Health Science and Policy, Icahn School of Medicine at Mount Sinai, New York, NY, USA; Department of Health Policy and Management, College of Public Health, University of Georgia, Athens, GA, USA; Department of Geography, University of Georgia, Athens, GA, USA; Department of Health Policy and Management, College of Public Health, University of Georgia, Athens, GA, USA; Department of Population Health Science and Policy, Icahn School of Medicine at Mount Sinai, New York, NY, USA; Institute for Translational Epidemiology, Icahn School of Medicine at Mount Sinai, New York, NY, USA; Department of Public Health Policy and Management, School of Global Public Health, New York University, New York, NY, USA

## Abstract

**Background:**

Young adult cancer survivors face medical financial hardships that may lead to delaying or forgoing medical care. This study describes the medical financial difficulties young adult cancer survivors in the United States experience in the post–Patient Protection and Affordable Care Act period.

**Method:**

We identified 1009 cancer survivors aged 18 to 39 years from the National Health Interview Survey (2015-2022) and matched 963 (95%) cancer survivors to 2733 control individuals using nearest-neighbor matching. We used conditional logistic regression to examine the association between cancer history and medical financial hardship and to assess whether this association varied by age, sex, race and ethnicity, and region of residence.

**Results:**

Compared with those who did not have a history of cancer, young adult cancer survivors were more likely to report material financial hardship (22.8% vs 15.2%; odds ratio = 1.65, 95% confidence interval = 1.50 to 1.81) and behavior-related financial hardship (34.3% vs 24.4%; odds ratio = 1.62, 95% confidence interval = 1.49 to 1.76) but not psychological financial hardship (52.6% vs 50.9%; odds ratio = 1.07, 95% confidence interval = 0.99 to 1.16). Young adult cancer survivors who were Hispanic or lived in the Midwest and South were more likely to report psychological financial hardship than their counterparts.

**Conclusions:**

We found that young adult cancer survivors were more likely to experience material and behavior-related financial hardship than young adults without a history of cancer. We also identified specific subgroups of young adult cancer survivors that may benefit from targeted policies and interventions to alleviate medical financial hardship.

Many people in the United States face financial difficulties and stress resulting from the high costs of medical care (1,2). Medical financial hardship, or “financial toxicity,” is an ongoing concern; approximately 137.1 million adults have reported some medical financial hardship in the past year (1). The prevalence of medical financial hardship varies across sociodemographic groups and populations (3); people with lower educational attainment or multiple health conditions or who live in rural areas are more likely to experience medical financial hardship (1,4-6). Among patients with cancer, the adverse consequences of medical financial hardship are more drastic, with approximately 50% of these patients facing personal financial burden associated with the disease and its treatment (7,8). Compared with individuals with no history of cancer, cancer survivors are more likely to face substantial out-of-pocket health-care expenditures and experience difficulties paying medical bills, endure financial distress, and delay or forgo medical care (3,5,9-12).

Many life-saving treatments and medications prescribed for patients with cancer are costly. For example, treatment for breast cancer, the most prevalent type of cancer among women in the United States, costs an average of $198 400 over 5 years for patients younger than 65 years of age and an average of $116 800 for patients 65 years of age and older (13). Cancer survivors incur additional expenses as a result of follow-up treatments or checkups. The accumulation of these cancer-related health-care expenditures imposes an enormous financial burden on both cancer survivors and their families, which often results in the depletion of family assets, increased stress on household finances, reduced adherence to needed medical care, and increased mortality (3,14,15).

Cancer survivors, regardless of age, are at higher risk of experiencing financial hardship for multiple reasons, such as impaired work ability (16,17), productivity losses (18,19), and decreased income (20,21) resulting from cancer treatment and its long-term effects. The impact of financial hardship, however, could be particularly challenging for cancer survivors during their young adulthood (18-39 years of age) (10,22) because this is the time when these young cancer survivors experience the transition from adolescence into adulthood and from being cancer patients into cancer survivors. Cancer diagnosis from childhood or during young adulthood could lead to disruptions in education and in work and family planning (23,24); these disruptions could further result in decreased earnings, financial struggles, change in health-care–seeking behaviors, and reductions in needed medical care (12). Also, young adults lack a long employment history and, as a result, are likely to have limited financial assets.

Nonetheless, existing literature has largely focused on either all-age adult cancer survivors together or separated by the age cutoff of 65 years (4,5,9,25-27) or adult survivors of adolescent and young adult cancer (a cancer diagnosis at 15-39 years of age) (10,28,29). The disruptive financial influence of a cancer diagnosis, treatment, and continued care on young adults (ie, those aged 18-39 years)—who are more vulnerable to medical financial hardship than other age groups—has yet to be examined. Using nationally representative data from the National Health Interview Survey (NHIS), we studied material, psychological, and behavioral financial hardships among young adult cancer survivors.

## Methods

### Data sources and study population

We used publicly available data from NHIS 2015-2020. The NHIS is an ongoing nationally representative cross-sectional survey that samples approximately 87 500 individuals from 35 000 households annually. Using a multistage probability design, the NHIS sample is designed to be representative of the civilian, noninstitutionalized population living in the 50 states and the District of Columbia (30). The survey provides information about the socioeconomic status, demographics, health conditions, and health-care access and utilization of the respondents.

A total of 73 059 adults 18 to 39 years of age participated in the survey from 2015 to 2022. We excluded 27 participants who did not answer the question, “Have you EVER been told by a doctor or other health professional that you had …[c]ancer or a malignancy of any kind?” or responded “Don’t know” or “Refused” to the question. We then excluded 163 participants who had nonmelanoma or unknown skin cancer only. We further excluded 1266 participants who had missing values on the financial hardship outcome variables and 2891 participants who had missing values on key covariates and survey design elements (eg, survey strata, clusters, weights). Our final study sample consisted of 68 712 participants, out of which 1009 were cancer survivors, defined as individuals who had been diagnosed with cancer (Supplementary Figure 1, available online).

### Outcome variables

Medical financial hardship was categorized into 3 domains: material conditions, psychological responses, and coping behaviors. This framework provides a multidimensional measure of medical financial hardship by capturing material conditions resulting from increased health-care expenses and reduced income (eg, difficulties in paying medical bills), psychological responses to treatment and care cost (eg, financial worry and distress), and consequent health-care–seeking behaviors (eg, delaying or forgoing care because of cost) (1,31,32). This approach has been adopted in previous studies, particularly in survey studies, with the benefit of summarizing information from multiple measures within the same domain of hardship (26,33). Material financial hardship was measured by the survey question, “In the past 12 months did you or anyone (in household) have problems paying or were unable to pay any medical bills?” It was categorized as a binary variable (Yes or No), with those who answered “Yes” considered to have material financial hardship. Psychological financial hardship was measured by the question, “If you get sick or have an accident, how worried are you that you will be able to pay your medical bills?” Those who answered “very worried” or “somewhat worried” were considered to have psychological financial hardship. Behavior-related financial hardship was measured by 5 binary variable (Yes or No) questions asking the respondents if they, in the past 12 months 1) had ever delayed medical care due to worry about cost, 2) had ever forgone medical care due to worry about cost, 3) were unable to afford prescription medicines when they needed them, 4) were unable to afford counseling or therapy when they needed it, and 5) were unable to afford dental care when they needed it (except for the year 2021). Those who answered “Yes” to any of the 5 questions were considered to have behavior-related financial hardship.

### Exposure variable

The exposure variable was having a history of cancer. We defined those who answered “Yes” to the survey question, “Have you EVER been told by a doctor or other health professional that you had …[c]ancer or a malignancy of any kind?” as cancer survivors and those who answered “No” as controls without a history of cancer.

### Covariates

We selected the following variables as potential confounders, based on the domain knowledge and existing literature (5,10,26,27). Individual-level demographic and clinical characteristics included age category at the time of the survey; self-reported sex; race and ethnicity; educational attainment; marital status; employment status; family income level as a percentage of the federal poverty level (FPL); health insurance coverage; residential region; and disease burden, calculated as the total number of 6 common chronic disease conditions. These conditions were based on a “Yes” response to the questions asking if they were ever told by a doctor that they had asthma, arthritis, cardiovascular disease (coronary heart disease, stroke, myocardial infarction, or angina pectoris), diabetes, respiratory disease (chronic obstructive pulmonary disease, emphysema, or chronic bronchitis), and hypertension.

### Statistical analysis

To account for confounding and reduce bias, we used nearest-neighbor matching without replacement to match between 1 and 3 controls without a history of cancer to each cancer survivor, based on the following variables: age category, sex, race and ethnicity, education, marital status, employment status, family income level as a percentage of FPL, health insurance coverage, residential region, and disease burden. The matching approach is often preferred over commonly applied regression analyses because it provides more robust estimates by allowing one to assess the systematic difference in observed characteristics between groups (34,35). We performed descriptive analyses to compare the distribution of baseline characteristics between cancer survivors and controls before and after matching. We reported standardized mean differences in baseline characteristics between cancer survivors and controls; differences of 10% or less were considered meaningfully balanced.

Considering the correlation of individuals in the matched sets, we then used conditional logistic regressions to estimate the association between having a history of cancer and financial hardship. The matched individuals retained their natural sampling weights for better balance and lower bias (36). In addition, to investigate whether this association varied according to sociodemographic factors, we conducted similar regression analyses for stratified groups by age, sex, race and ethnicity, and residential region. All the analyses, unless specified, accounted for survey design elements, including survey strata, clusters, and sampling weights to generate population-level estimates. We used SAS, version 9.4, statistical analysis software (SAS Institute LP, Cary, NC) to perform all analyses and R, version 4.2.1 (R Foundation for Statistical Computing, Vienna, Austria) and Excel, version 2016 (Microsoft Corp, Redmond, WA) to create the figures. All tests were 2-sided, with *P*  less than .05 considered statistically significant.

## Results

### Population characteristics

Of 1009 young adult cancer survivors, 963 (95%) were matched to 2733 controls. Table 1 provides the distributions of baseline characteristics between cancer survivors and controls before and after matching. Before matching, the 2 groups of participants differed systematically. After matching, the characteristics of cancer survivors and their controls were similar, with standardized differences less than 10% for all variables.

**Table 1.** Baseline characteristics of young adults with and without a history of cancer, before and after matching

**Variable**	Before matching			After matching		
	Cancer survivors (n = 1009)	Controls (n = 67 703)	Standardized mean difference	Cancer survivors (n = 963)	Controls (n = 2733)	Standardized mean difference
Age, No. (weighted %), y						
18-25	111 (13.1)	19 363 (35.8)	0.45	102 (12.7)	286 (13.9)	<0.01
26-39	898 (86.9)	48 340 (64.2)	0.45	861 (87.3)	2447 (86.1)	<0.01
Female sex, No. (weighted %)	719 (66.5)	35 813 (50.1)	0.39	689 (66.5)	1949 (68.6)	0.01
Race and ethnicity, No. (weighted %)						
Non-Hispanic White	742 (70.6)	39 841 (56.3)	0.31	718 (71.6)	2062 (74.5)	0.02
Non-Hispanic Black	74 (7.9)	8048 (13.2)	0.15	68 (7.4)	188 (7.5)	0.01
Hispanic	140 (16.8)	13 133 (21.4)	0.15	130 (16.4)	359 (13.8)	0.01
Other^a^	53 (4.7)	6681 (9.1)	0.18	47 (4.5)	124 (4.1)	0.02
Education, No. (weighted %)						
Less than high school	127 (14.7)	7171 (12.2)	0.06	114 (13.7)	300 (11.8)	0.03
High school graduate	157 (17.3)	13 601 (23.2)	0.12	144 (16.7)	399 (15.8)	0.01
College or higher	725 (68.0)	46 931 (64.6)	0.06	705 (69.6)	2034 (72.4)	0.03
Marital status, No. (weighted %)						
Married/living with a partner	556 (62.0)	32 696 (49.8)	0.14	525 (61.8)	1499 (61.0)	0.01
Never married/others	453 (38.0)	35007 (50.2)	0.14	438 (38.2)	1234 (39.0)	0.01
Employment, No. (weighted %)						
Full time	539 (51.0)	41 221 (57.5)	0.15	522 (51.5)	1618 (58.3)	0.1
Part-time	184 (17.7)	11 625 (18.4)	0.03	175 (17.6)	440 (17.0)	0.06
Unemployed	286 (31.3)	14 857 (24.1)	0.15	266 (30.9)	675 (24.7)	0.07
Family income level as a percentage of the federal poverty level, No. (weighted %)						
<200%	417 (41.0)	23 563 (33.0)	0.13	390 (39.9)	1073 (35.9)	0.03
200%-399%	266 (25.7)	20 179 (30.6)	0.08	256 (26.0)	733 (29.0)	0.01
≥400%	326 (33.3)	23 961 (36.4)	0.07	317 (34.0)	927 (35.1)	0.02
Health insurance, No. (weighted %)						
Private and other	630 (62.5)	48 089 (69.1)	0.18	611 (63.4)	1794 (65.1)	0.05
Medicaid and other public insurance	248 (23.4)	10 000 (15.7)	0.25	231 (23.3)	634 (23.2)	0.02
Uninsured	131 (14.1)	9614 (15.2)	0.04	121 (13.3)	305 (11.6)	0.04
Region of residence, No. (weighted %)						
Northeast	137 (13.4)	10 170 (16.3)	0.04	132 (13.2)	379 (16.0)	<0.01
Midwest	260 (25.8)	15 314 (21.8)	0.07	246 (25.1)	697 (25.7)	<0.01
South	331 (35.4)	23 926 (36.9)	0.05	319 (35.9)	902 (34.1)	<0.01
West	281 (25.4)	18 293 (25.0)	0.02	266 (25.8)	755 (24.2)	<0.01
Disease burden,^b^ No. (weighted %)						
None	526 (51.0)	48 164 (71.8)	0.4	526 (53.8)	1561 (57.4)	0.05
1	274 (28.0)	15 584 (22.7)	0.1	268 (29.0)	775 (29.5)	0.01
2	136 (14.0)	3130 (4.3)	0.31	119 (12.3)	300 (10.1)	0.04
≥3	73 (7.0)	825 (1.1)	0.3	50 (4.8)	97 (3.0)	0.08
Time since diagnosis, weighted mean (SD), y	10.0 (0.5)			10.2 (0.3)		

a “Other” includes Asian, American Indian and Alaska Native, and other race or ethnicity.

b *Disease burden* was defined as the number of the following 6 common chronic conditions, based on whether they were ever told by a physician that they had asthma, arthritis, cardiovascular disease (coronary heart disease, stroke, myocardial infarction, or angina pectoris), diabetes, respiratory disease (chronic obstructive pulmonary disease, emphysema, or chronic bronchitis), and hypertension.

### Association of a history of cancer with financial hardship

**Figure 1.**
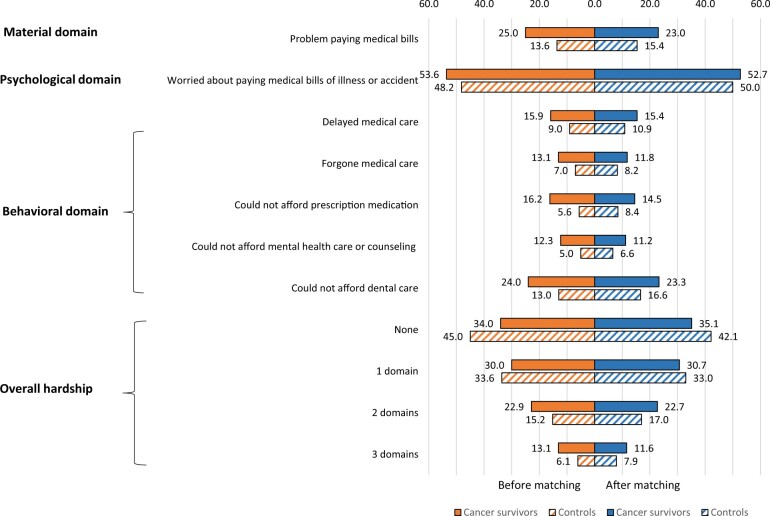
Proportion of young adult cancer survivors and control individuals reporting material, psychological, and behavioral financial hardship before and after matching.

**Figure 2.**
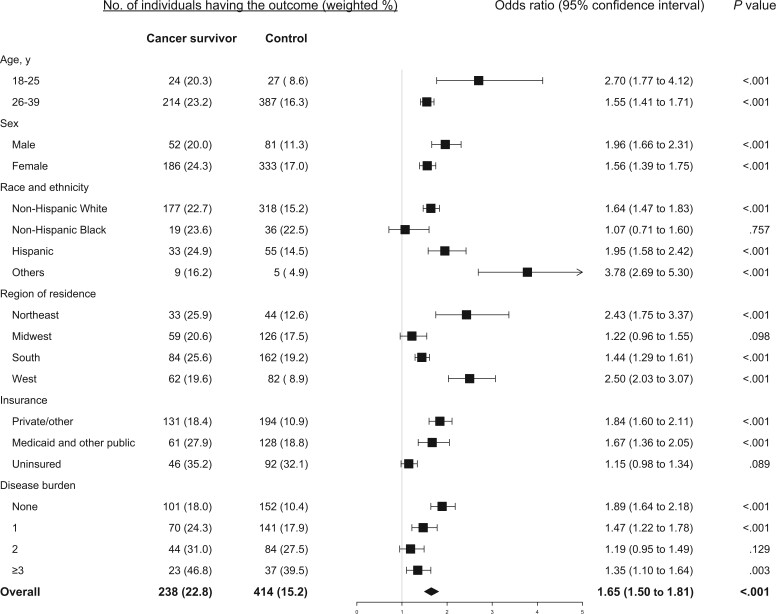
Estimated association between history or cancer and material financial hardship overall and stratified by age, sex, race and ethnicity, residential region, health insurance coverage, and disease burden.

**Figure 3.**
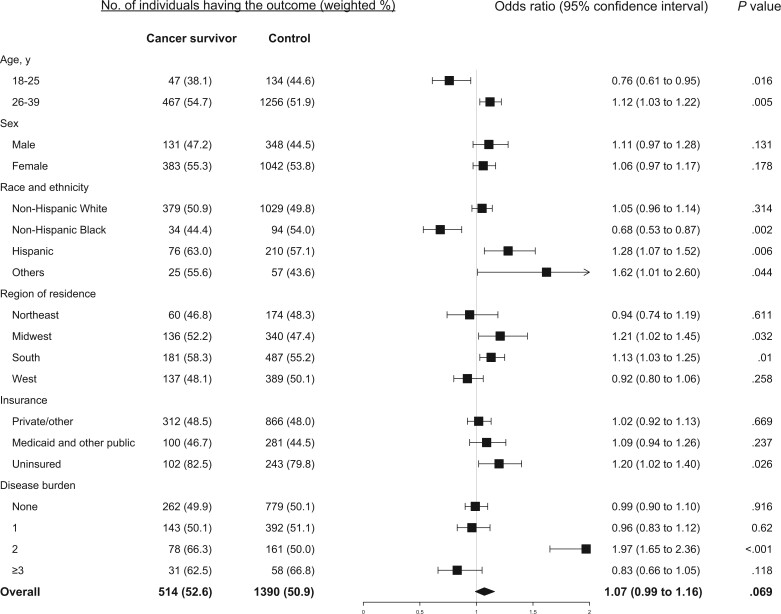
Estimated association between history of cancer and psychological financial hardship overall and by age, sex, race and ethnicity, residential region, health insurance coverage, and disease burden.

**Figure 4.**
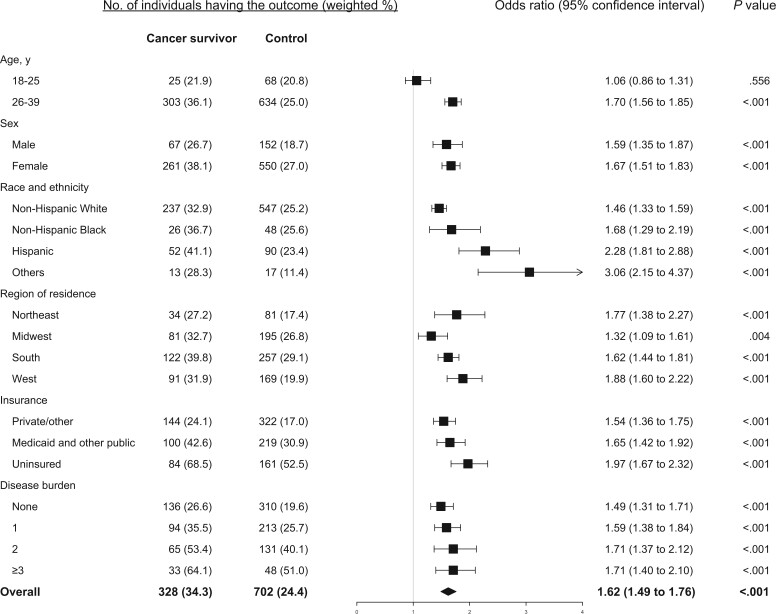
Estimated association between history of cancer and behavioral financial hardship, stratified by age, sex, race and ethnicity, residential region, health insurance coverage, and disease burden.

Among cancer survivors, 22.8%, 52.6%, and 34.3% reported having material, psychological and behavior-related financial hardship, respectively, compared with 15.2%, 50.9%, and 24.4% among the matched controls. Also compared with their counterparts, cancer survivors were more likely to report having financial hardship in 2 domains (21.8% vs 16.2%, *P* = .002) and in all 3 domains (11.7% vs 7.9%, *P* = .002). The detailed response to each measure in the 3 domains for cancer survivors and their controls is provided in Figure 1. Compared with those with no history of cancer, cancer survivors had significantly higher odds of material financial hardship (odds ratio [OR] = 1.65, 95% confidence interval [CI] = 1.50 to 1.81) and behavior-related financial hardship (OR = 1.62, 95% CI = 1.49 to 1.76) but not psychological financial hardship (OR = 1.07, 95% CI = 0.99 to 1.16) (Figures 2-4).

### Association of a history of cancer with financial hardship, stratified by age, sex, race and ethnicity, residential region, health insurance status, disease burden, and cancer type

Compared with those who did not have a history of cancer, cancer survivors 26 to 39 years of age consistently had significantly increased odds of reporting material (Figure 2), psychological (Figure 3), and behavioral (Figure 4) financial hardship, while cancer survivors 18 to 25 years of age had significantly lower odds (OR = 0.70, 95% CI = 0.61 to 0.82) of reporting psychological financial hardship. Both male and female cancer survivors had significantly increased odds of reporting material (Figure 2) and behavioral financial hardship (Figure 4), but not psychological financial hardship (Figure 3).

Cancer survivors of all racial and ethnic groups had significantly increased odds of reporting behavioral (Figure 4) and material financial hardship (Figure 2, except the Black group). For psychological financial hardship, increased odds were significant only in Hispanic (OR = 1.28, 95% CI = 1.07 to 1.52) and other racial or ethnic (OR = 1.62, 95% CI = 1.01 to 2.60) young adult cancer survivors (Figure 3).

Young adult cancer survivors from the Northeast or West region had significantly higher odds of reporting material (Figure 2) and behavioral financial hardship (Figure 4) but not psychological financial hardship (Figure 3). In contrast, cancer survivors residing in the Midwest or South region were more likely to report psychological financial hardship, with an estimated OR = 1.21 (95% CI = 1.02 to 1.45) and OR = 1.13 (95% CI = 1.03 to 1.25), respectively (Figure 3). They also had increased odds of reporting behavioral financial hardship (Figure 4).

Compared with their counterparts, young adult cancer survivors, regardless of their insurance coverage and disease burden, had significantly higher odds of reporting behavioral financial hardship, particularly for those uninsured and those who had multiple chronic diseases (Figure 4).

## Discussion

Using nationally representative NHIS data from 2015 to 2022, we conducted a matching analysis to examine the association between cancer history and medical financial hardship, measured in the material, psychological, and behavioral domains, by comparing young adult cancer survivors with their matched counterparts with no history of cancer. We found that having a cancer history was significantly associated with financial hardship in the material and behavioral domains. Compared with their matched controls, young adult cancer survivors had higher odds of reporting financial hardship in the material and behavioral domains. They were also more likely to report financial hardship in multiple domains. The magnitude and direction of our findings are in line with a recent study using the 2014 to 2016 NHIS data for cancer survivors of all ages as well as for the age group of 18- to 49-year-olds (5). These findings suggest an opportunity for the health-care team to initiate cost-related discussions proactively and assist patients with financial challenges associated with their cancer treatment and care.

We did not find a significant association between a history of cancer and psychological financial hardship. This null finding is congruent with a recent study by Lu et al. (10) that focused on adult survivors with a cancer diagnosis when they were adolescents or young adults, in contrast to an earlier study by Zheng et al. (5). The lack of statistically significant associations does not preclude the existence of such an association. There are notable differences between the study by Zheng et al. and ours. In the work by Zheng and colleagues, psychological financial hardship was measured by both worries about medical bills for normal care and care for accidents or being sick, while only a question on worries about medical bills for accidents or being sick was available in the NHIS in later years. In addition to the differences in how psychological financial hardship was defined, other possible explanations of different findings include the study time window (eg, 2013-2016 vs 2015-2022) and analytic sample (eg, adults of all ages vs young adults 18-39 years of age). Moreover, depending on whether the survey design was accounted for in the analysis, the findings may be inferred to the target population or limited to the analytic sample. In our study, we incorporated the survey design elements in the regression analysis for the matched sample of young adult cancer survivors, which allowed us to draw inferences at the national population level. Nevertheless, to further evaluate the relative impact of a history of cancer on different domains of financial hardship, particularly on the psychological domain, additional evidence, with comprehensive measures of cancer survivors’ perception of worry and detailed information about their personal finances, family support, insurance status, and cancer-related treatments, duration, and costs, is needed.

We also found that the impact of a history of cancer on young adult cancer survivors’ financial hardship varied by several sociodemographic and health factors. For example, compared with their counterparts, cancer survivors with no insurance coverage or who have multiple chronic diseases were more than likely to experience behavioral financial hardship. Also, Hispanic and other racial and ethnic young adult cancer survivors were more likely to report psychological financial hardship than non-Hispanic White young adult cancer survivors in addition of the other 2 domains. This finding may be explained by differences in their health insurance status, where the proportion of those who were uninsured was 18.8% in the Hispanic group and 14.4% in the other racial and ethnic group compared with 10.5% among those in the non-Hispanic White group in our study. Our results suggest that targeted interventions to improve health insurance coverage and access to health care among Hispanic and those in the other racial and ethnic young adult cancer survivors may reduce their financial burden resulting from medical bills and related stress and concerns.

Our results also show that cancer survivors in the South and Midwest were more likely to report psychological financial hardship. Material financial hardship has been demonstrated to be associated with a lack of health insurance coverage (26,37), which may further perpetuate psychological financial hardship and increase the worry about medical bills. It is noteworthy that the uninsured rates in our study sample were 3.5% and 8.1% for the Northeast and West, respectively, while the rates were 14.9% and 16.6% for the Midwest and South, respectively. The Patient Protection and Affordable Care Act (ACA), enacted in 2010, attempted to reduce the size of the uninsured population through a continuum of affordable coverage provisions (38). One of the ACA’s provisions was to allow young adults to remain in their parent’s private health insurance plans as dependents until they reach age 26 years. Medicaid expansion under the ACA has been shown to be associated with improved health insurance coverage and overall survival among young adults with cancer (39,40). It may also mitigate the financial hardship younger cancer survivors fact. The adoption of Medicaid expansion to cover poor and near-poor individuals with incomes up to 138% of the federal poverty level, however, is a state-level decision. Among the 11 states that have not expanded Medicaid for these populations, 10 were in the South and Midwest (41). Understanding the impact of these ACA provisions on medical financial hardship among young adult cancer survivors, particularly by state and region of residence, would be an important topic for future research.

Our study has some limitations. First, answers to questions in the NHIS were self-reported, which is subject to recall bias (42). Second, due to the cross-sectional nature of NHIS design, we were unable to identify the exact time order of the experience of financial hardship and cancer diagnosis, particularly when they were reported in the same year, and were thus unable to establish a causal relationship between cancer history and financial hardship. Third, considering the consistency of survey questions over the study years, psychological financial hardship was measured only by the respondents’ worry about medical bills if they should get sick or have an accident but not about normal care. Finally, we did not examine the impact of financial hardship by cancer types due to concerns about sample size; also, the NHIS lacks detailed information about cancer diagnoses, such as disease stage, duration of illness, and treatment history, and health condition. We considered comorbidities in the matching process, however, which may ameliorate some of the concerns about the lack of detailed cancer information.

Compared with their counterparts with no history of cancer, young adult cancer survivors 18 to 39 years of age had an elevated risk of experiencing medical financial hardship, particularly material and behavioral financial hardships. The patterns of material, psychological, and behavioral medical financial hardship vary by age, sex, race and ethnicity, and residential region. Our study highlights the importance of studying this age group of cancer survivors specifically. Findings also suggest that interventions that reduce medical financial hardship in young adult cancer survivors should focus on improving health insurance coverage and specific risk groups.

## Supplementary Material

pkae007_Supplementary_Data

## Data Availability

The NHIS data are publicly available and can be found at the website of the Centers for Disease Control and Prevention (https://www.cdc.gov/nchs/nhis/data-questionnaires-documentation.htm).
