# Supplementary material for: Medical financial hardship between young adult cancer survivors and matched individuals without cancer in the United States
Source: JNCI Cancer Spectr. 2024 Feb 14;8(2):pkae007. doi: 10.1093/jncics/pkae007 (PMC10903972; doi:10.1093/jncics/pkae007)
Supplement: pkae007_Supplementary_Data [file pkae007_supplementary_data.pdf]

Supplementary Material

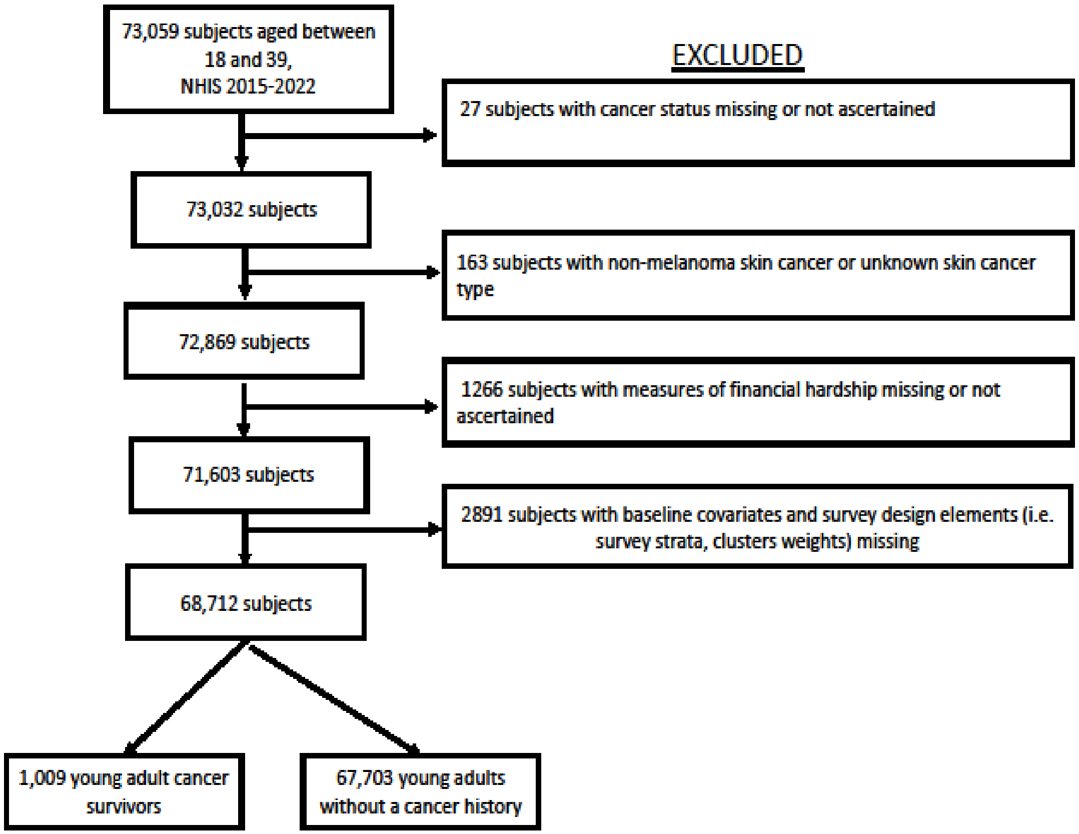

Supplementary Figure 1. Flow chart of the analytic cohort before matching
